# Genetic Insights Into Cardiomyopathy With Restrictive Phenotype: Implications for Diagnosis and Personalized Care

**DOI:** 10.1002/mgg3.70323

**Published:** 2026-10-25

**Authors:** Nianwei Zhou, Yi Sun, Xuemei Wei, Wenwen Liang, Fangmin Meng, Zhengdan Ge, Minmin Sun, Xiaotong Cui, Shuning Zhang, Cuizhen Pan, Junbo Ge, Xianhong Shu

**Affiliations:** ^1^ Department of Echocardiography, Zhongshan Hospital Fudan University Shanghai China; ^2^ Shanghai Institute of Cardiovascular Disease Shanghai China; ^3^ Shanghai Institute of Medical Imaging Shanghai China; ^4^ Department of Cardiology, Zhongshan Hospital Fudan University Shanghai China

**Keywords:** cardiomyopathy, echocardiography, genotype, restrictive phenotype

## Abstract

**Background:**

Cardiomyopathy with a restrictive phenotype is uncommon, genetically heterogeneous, and difficult to distinguish from advanced hypertrophic or dilated cardiomyopathy. We aimed primarily to determine the diagnostic yield and spectrum of pathogenic or likely pathogenic variants among patients with an echocardiographically defined restrictive phenotype; genotype‐imaging and survival comparisons were prespecified exploratory analyses.

**Methods:**

We screened 568 patients with cardiomyopathy who underwent whole‐exome sequencing and comprehensive echocardiography. Thirty‐three met the operational definition of restrictive phenotype. Variants were classified. Genotype‐imaging associations and survival were analyzed exploratorily.

**Results:**

Among the 568 patients, 33 exhibited a restrictive phenotype: 7 had restrictive cardiomyopathy (RCM), 20 had hypertrophic cardiomyopathy with a restrictive phenotype (HRCM), and 6 had dilated cardiomyopathy with a restrictive phenotype (HDRCM). Variant carriers had a smaller aortic sinus diameter. In addition, MYBPC3 variant carriers had greater ventricular wall thickness and more frequent septal hypertrophy. Both the HRCM and HDRCM groups showed hypertrophic features, with interventricular septal hypertrophy occurring more frequently in the HDRCM group. The estimated 5‐year survival was 54.5%, and survival did not differ significantly according to genetic‐variant status.

**Conclusions:**

Pathogenic or likely pathogenic variants were frequent in this single‐center cohort with restrictive physiology and were associated with selected imaging features. These exploratory findings support integrated genetic and echocardiographic evaluation.

## Graphical abstract

Among 568 patients with cardiomyopathy, 33 exhibited a restrictive phenotype, including 7 with RCM, 20 with HRCM, and 6 with HDRCM. Pathogenic or likely pathogenic variants were identified in 24 patients (72.7%). Variant carriers had a smaller aortic sinus diameter than non‐carriers (30.79 ± 3.48 vs. 34.22 ± 4.44 mm; nominal *p* = 0.026), while MYBPC3 was the most frequently involved gene and was associated with greater LV wall thickness and more frequent septal hypertrophy. Estimated 5‐year survival was 54.5%, with no significant difference according to P/LP variant status. These exploratory findings support integrating genetic testing with echocardiographic phenotyping to improve etiologic diagnosis.

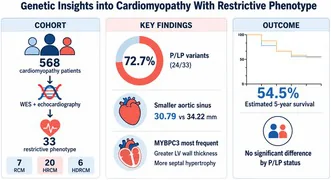

## 1 Introduction

Restrictive cardiomyopathy (RCM) is a rare and clinically challenging form of cardiomyopathy associated with high morbidity and mortality (Zheng et al. 2022). Because of its low prevalence, phenotypic heterogeneity, and limited treatment options, RCM has long been regarded as an orphan disease. Nonetheless, recent therapeutic advances, particularly in cardiac amyloidosis, have raised new hope for patients and families affected by restrictive myocardial disorders (Rapezzi et al. 2022).

Cardiomyopathies represent a heterogeneous spectrum of myocardial diseases with diverse genetic determinants and clinical presentations. Among these entities, RCM is the least prevalent subtype and is defined by restrictive ventricular physiology, characterized by impaired diastolic filling in the presence of preserved or near‐preserved systolic function (Hayashi et al. 2018). The diagnosis of RCM is often complicated by substantial phenotypic overlap with hypertrophic cardiomyopathy (HCM) and dilated cardiomyopathy (DCM). Moreover, restrictive features may also be observed in subsets of patients with HCM and DCM, further blurring conventional phenotypic boundaries and complicating clinical assessment and management (Elliott et al. 2008; Kubo et al. 2007). Accordingly, a clearer understanding of the genetic basis of restrictive phenotypes across the cardiomyopathy spectrum is critical for elucidating disease mechanisms, improving phenotypic classification, and advancing precision medicine (Caleshu et al. 2011).

The genetic architecture of cardiomyopathy with restrictive physiology remains incompletely defined, and prior studies have often focused on primary RCM rather than restrictive physiology across conventional cardiomyopathy categories. Our primary objective was therefore to determine the diagnostic yield and spectrum of pathogenic or likely pathogenic (P/LP) variants in patients with an echocardiographically defined restrictive phenotype. Secondary exploratory objectives were to compare imaging features by genotype and phenotype and to describe survival. We hypothesized that restrictive physiology would identify a genetically enriched subgroup with genotype‐associated imaging patterns.

## 2 Methods

### 2.1 Study Population

A total of 568 patients with cardiomyopathy were enrolled between 2019 and 2021, including 389 with HCM, 172 with DCM, and 7 with primary RCM. The original HCM and DCM diagnoses were assigned using guideline‐based morphologic criteria before restrictive physiology was assessed. A restrictive phenotype was operationally defined by evidence of elevated filling pressure and restrictive filling together with biatrial enlargement, after exclusion of significant valvular disease, pericardial disease, and constrictive physiology. The prespecified echocardiographic criteria incorporated the transmitral E/A ratio, E‐wave deceleration time, and average E/e' ratio (> 14). Patients with pre‐existing HCM or DCM who met these criteria remained classified as HRCM or HDRCM, respectively, rather than primary RCM; this approach was intended to avoid relabeling advanced HCM or DCM as primary RCM. Follow‐up continued until death or the last known contact. Patients alive at the last contact were right‐censored on that date. The protocol was approved by the Ethics Committee of Zhongshan Hospital, Fudan University (No. B2016‐016(2)R). Written informed consent for participation and genetic testing was obtained from all participants or their legal guardians.

### 2.2 Echocardiography

All patients underwent comprehensive transthoracic echocardiography performed by experienced sonographers. Image acquisition and measurements were conducted in accordance with the recommendations of the American Society of Echocardiography. Standard echocardiographic parameters included left ventricular dimensions and wall thickness, left ventricular ejection fraction, indices of diastolic function, right ventricular dimensions and function, and tricuspid regurgitation velocity. To further characterize regional myocardial morphology, the left ventricular wall was divided into 16 segments on the basis of two‐dimensional short‐axis views. Segmental wall thickness was measured individually; segments with a wall thickness > 12 mm were defined as hypertrophic and assigned a Score of 1, whereas segments with a wall thickness ≤ 12 mm were assigned a score of 0. The sum of the segmental scores was used to reflect the extent and distribution of left ventricular hypertrophy.

### 2.3 Genotyping

Whole‐exome sequencing was performed in all participants. Sequencing, data processing, and variant interpretation followed protocols previously reported by our group (Zhou et al. 2023, 2022). Reads were aligned to GRCh37/hg19, followed by variant calling, annotation, and filtering. Rare coding and canonical splice‐site variants were prioritized according to population frequency, predicted consequence, phenotype consistency, segregation evidence when available, and published or curated evidence. Variants were classified as pathogenic or likely pathogenic according to ACMG/AMP criteria, with the applied evidence codes documented during multidisciplinary review.

### 2.4 Statistical Analysis

All statistical analyses were performed using SPSS version 24.0 (IBM Corp., Armonk, NY, USA). Continuous variables are presented as mean ± SD, and categorical variables as *n* (%). Categorical variables were compared using Pearson's chi‐square test or Fisher's exact test, as appropriate. Continuous variables were compared using an independent‐samples *t*‐test for two groups and the Kruskal‐Wallis test for three groups after assessment of distributional assumptions. Survival was estimated using the Kaplan–Meier method; patients alive at the last contact were right‐censored, and groups were compared using the log‐rank test. Genotype‐imaging and segment‐level comparisons were exploratory. Because of the small cohort and correlated echocardiographic measures, no formal adjustment for multiple comparisons was applied; exact two‐sided *p*‐values are reported, and findings with nominal *p* < 0.05 should be interpreted as hypothesis‐generating rather than confirmatory.

## 3 Results

### 3.1 Demographic Characteristics

Among 568 patients with cardiomyopathy, including 389 with HCM, 172 with DCM, and 7 with RCM, 33 were identified as having a restrictive phenotype. This subgroup included 20 patients with HCM and restrictive phenotype (HRCM) and 6 patients with DCM and restrictive phenotype (HDRCM), in addition to the 7 patients with RCM. Male predominance was observed, particularly in the HRCM and HDRCM groups. Age distribution did not differ significantly among the three groups (Table S1).

### 3.2 Genotype–Phenotype Associations

Pathogenic or likely pathogenic variants were identified in 24 of 33 patients (72.7%) with restrictive phenotype (Figure 1). Compared with non‐carriers, carriers had a smaller aortic sinus dimension (30.79 ± 3.48 vs. 34.22 ± 4.44 mm; nominal *p* = 0.026), whereas other conventional echocardiographic parameters were similar (Table 1; Table S2). This association was exploratory and was not adjusted for multiple comparisons. MYBPC3 was the most frequently involved gene and occurred predominantly in patients classified as HRCM; one additional carrier had HDRCM with a concurrent DES variant. MYBPC3 carriers had greater anteroseptal and maximum LV wall thickness and more frequent septal hypertrophy than carriers of other genotypes (Table 2; Table S3). TTR and LAMP2 variants occurred in patients with overlapping hypertrophic/restrictive or dilated/restrictive features.

**FIGURE 1:**
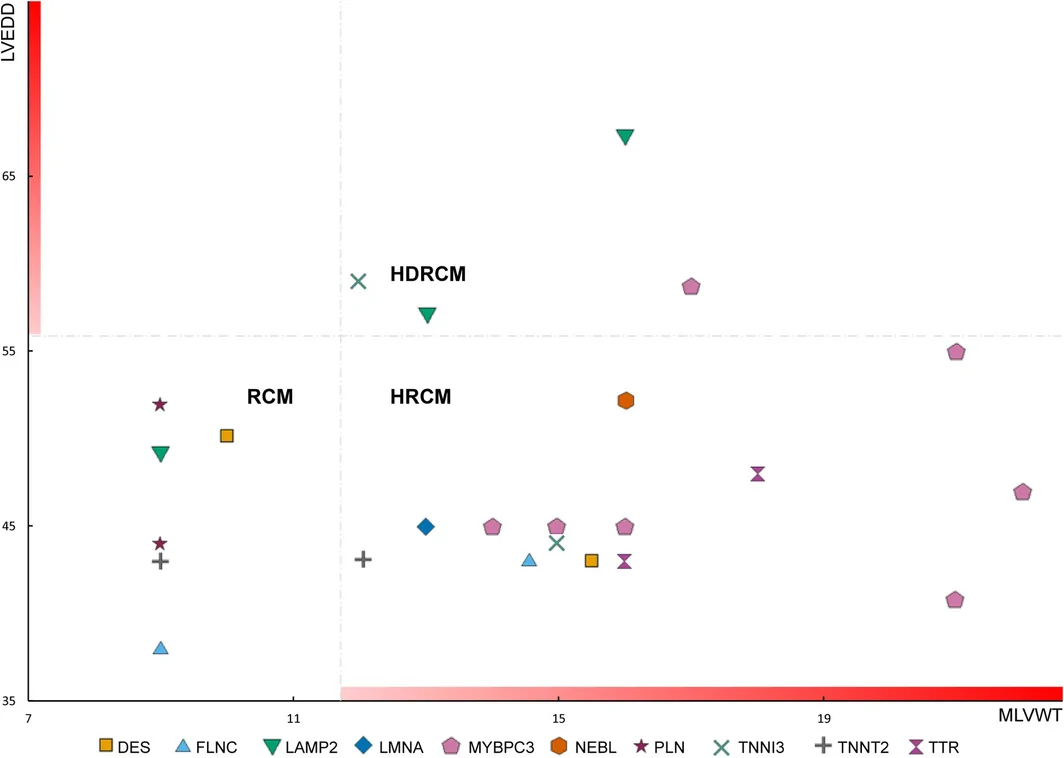
Distribution of genes with reportable variants among patients with a restrictive phenotype. Each point represents one patient and is positioned by maximum left ventricular wall thickness (MLVWT) and LV end‐diastolic diameter (LVEDD).

**TABLE 1:** Baseline characteristics of patients with restrictive phenotype according to genetic status.

Characteristic	Entire cohort (*n* = 33)	Genotype‐negative (*n* = 9)	Genotype‐positive (*n* = 24)	*p*
Age (years)	52.67 ± 16.76	53.33 ± 15.94	52.42 ± 17.38	0.891
Male, *n* (%)	21 (63.64)	5 (55.56)	16 (66.67)	0.569
Body mass index (kg/m^2^)	24.7 ± 6.9	25.3 ± 6.5	24.2 ± 7.7	0.467
Aortic sinus diameter (mm)	31.73 ± 4.00	34.22 ± 4.44	30.79 ± 3.48	0.026
Left atrial diameter (mm)	48.88 ± 7.48	47.56 ± 6.39	49.38 ± 7.92	0.542
LV end‐diastolic diameter (mm)	47.7 ± 7.43	47.22 ± 9.76	47.88 ± 6.6	0.826
LV end‐systolic diameter (mm)	35.12 ± 8.21	33.22 ± 9.54	35.83 ± 7.77	0.425
Anteroseptal wall thickness (mm)	13.27 ± 3.92	14.44 ± 4.39	12.83 ± 3.73	0.300
Posterior wall thickness (mm)	12.61 ± 3.74	14.00 ± 5.00	12.08 ± 3.12	0.194
Maximum LV wall thickness (mm)	14.61 ± 5.04	16.44 ± 6.89	13.92 ± 4.12	0.204
RV hypertrophy, *n* (%)	18 (54.55)	6 (66.67)	12 (50.00)	0.408
PASP (mmHg)	42.73 ± 11.46	39.78 ± 10.5	43.83 ± 11.82	0.374
LVEF (%)	47.18 ± 7.92	48.22 ± 6.12	46.79 ± 8.59	0.651
TAPSE (mm)	13.0 ± 3.1	12.78 ± 4.27	13.08 ± 2.65	0.806
Abnormal myocardial echogenicity, *n* (%)	17 (51.52)	5 (55.56)	12 (50.00)	0.784
LV systolic dysfunction, *n* (%)	29 (87.88)	7 (77.78)	22 (91.67)	0.399
RV systolic dysfunction, *n* (%)	29 (87.88)	7 (77.78)	22 (91.67)	0.399
E/e' ratio	19.4 ± 5.5	18.9 ± 4.7	19.8 ± 5.9	0.434

*Note:* Data are presented as mean ± SD or *n* (%).

Abbreviations: LV, left ventricular; LVEF, left ventricular ejection fraction; PASP, pulmonary artery systolic pressure; RV, right ventricular; TAPSE, tricuspid annular plane systolic excursion.

**TABLE 2:** Segmental hypertrophy among patients with reportable genetic variants: MYBPC3 versus non‐MYBPC3 variants.

Characteristic	MYBPC3 variant (*n* = 7)	Non‐MYBPC3 variants (*n* = 17)	*p*
Basal anterior (S1)	6 (85.71)	8 (47.06)	0.063
Basal anteroseptal (S2)	7 (100.00)	8 (47.06)	0.008
Basal inferoseptal (S3)	7 (100.00)	8 (47.06)	0.008
Basal inferior (S4)	6 (85.71)	8 (47.06)	0.077
Basal inferolateral (S5)	6 (85.71)	8 (47.06)	0.077
Basal anterolateral (S6)	6 (85.71)	8 (47.06)	0.077
Mid anterior (S7)	6 (85.71)	8 (47.06)	0.077
Mid anteroseptal (S8)	7 (100.00)	8 (47.06)	0.012
Mid inferoseptal (S9)	7 (100.00)	8 (47.06)	0.012
Mid inferior (S10)	6 (85.71)	8 (47.06)	0.077
Mid inferolateral (S11)	6 (85.71)	8 (47.06)	0.077
Mid anterolateral (S12)	6 (85.71)	8 (47.06)	0.077
Apical anterior (S13)	6 (85.71)	8 (47.06)	0.087
Apical septal (S14)	7 (100.00)	8 (47.06)	0.015
Apical inferior (S15)	6 (85.71)	8 (47.06)	0.087
Apical lateral (S16)	6 (85.71)	9 (52.94)	0.144
Basal (S1‐S6)	5.43 ± 1.51	2.83 ± 3.09	0.033
Mid‐cavity (S7‐S12)	5.43 ± 1.51	2.83 ± 3.09	0.041
Apical (S13‐S16)	3.57 ± 1.13	1.94 ± 2.01	0.061
Anterior (S1 + S7 + S13)	2.57 ± 1.13	1.41 ± 1.54	0.068
Anteroseptal (S2 + S8 + S14)	3.00 ± 0.00	1.41 ± 1.54	0.009
Inferoseptal (S3 + S9)	2.00 ± 0.00	0.94 ± 1.03	0.008
Inferior (S4 + S10 + S15)	2.57 ± 1.13	1.41 ± 1.54	0.076
Inferolateral (S5 + S11)	1.71 ± 0.76	0.94 ± 1.03	0.077
Anterolateral (S6 + S12 + S16)	2.57 ± 1.13	1.47 ± 1.50	0.087

*Note:* Data are presented as *n* (%) or mean ± SD. Variants refer to pathogenic or likely pathogenic variants.

No significant differences were observed among the three groups in left ventricular systolic function, right ventricular systolic function, or pulmonary artery pressure. Left ventricular wall thickness was comparable between the HRCM and HDRCM groups. However, right ventricular hypertrophy was more prominent in the HRCM and HDRCM groups than in the RCM group. Segmental analysis showed that hypertrophy in HRCM was distributed relatively uniformly throughout the left ventricle, whereas HDRCM showed a similar pattern with a greater predominance of interventricular septal involvement (83.33%). The comparable frequencies of right and left ventricular hypertrophy in both groups suggest a hypertrophic remodeling pattern distinct from that of typical HCM (Figures 2 and 3).

**FIGURE 2:**
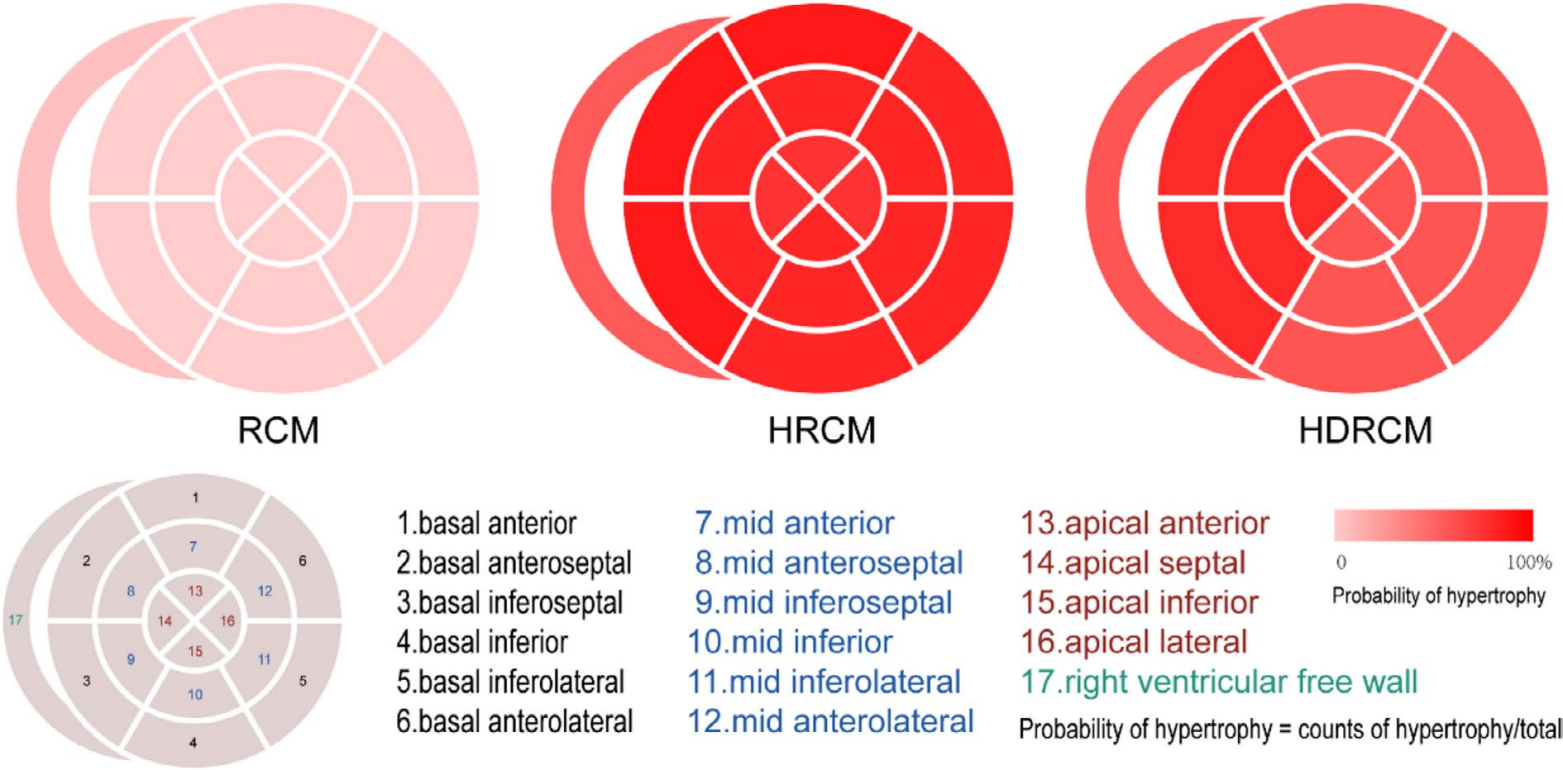
Hypertrophic pattern and right ventricular characteristics among groups. Heat map of the proportion of patients with hypertrophy in each LV segment and the RV free wall.

**FIGURE 3:**
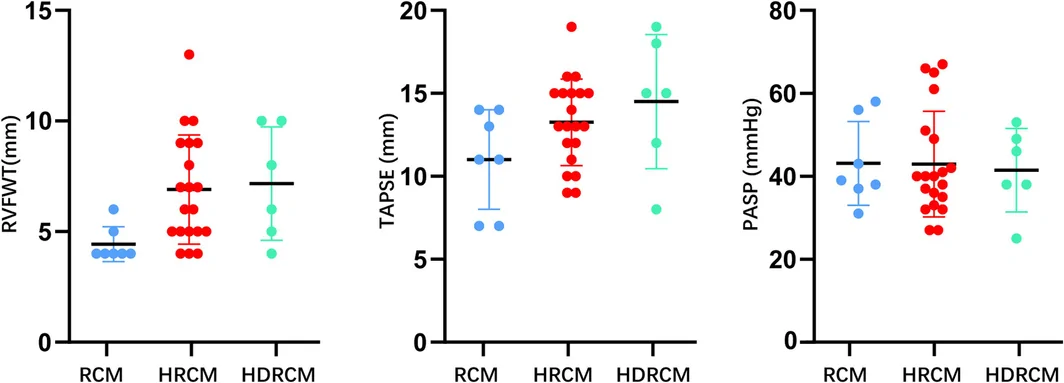
Individual‐patient dot plots showing the median and interquartile range for RV free‐wall thickness (RVFWT), TAPSE, and PASP across the RCM, HRCM, and HDRCM groups.

### 3.3 Clinical Follow‐Up

The estimated 5‐year survival was 54.5%. Survival did not differ significantly by P/LP variant status (Figure 4). Numbers at risk at prespecified time points have been added beneath the Kaplan–Meier curves. Multiple‐organ failure was the most common recorded cause of death, followed by sudden cardiac death. Patient‐level survival status, age at death or transplantation, follow‐up duration, and censoring status are provided in Table S1.

**FIGURE 4:**
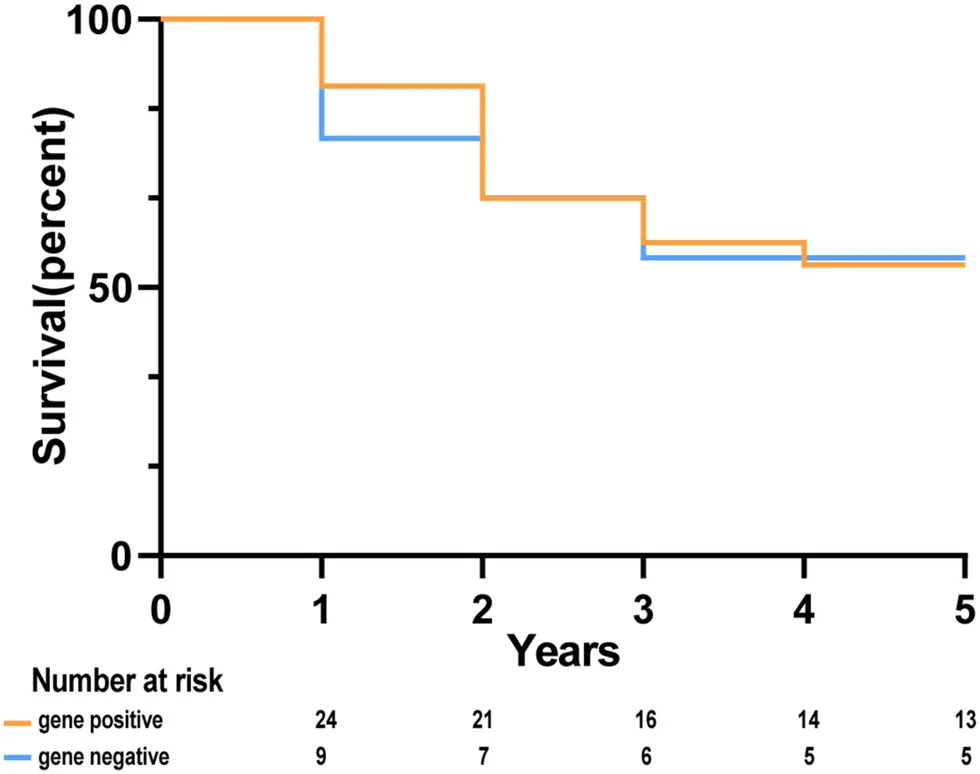
Kaplan–Meier survival curves with numbers at risk.

## 4 Discussion

A restrictive phenotype is an uncommon but clinically important manifestation across the cardiomyopathy spectrum and remains difficult to classify using conventional morphology alone (Wu et al. 2015). By integrating whole‐exome sequencing with echocardiographic phenotyping, we found a high yield of reportable genetic variants and identified distinct imaging characteristics in this cohort. These findings suggest that a restrictive phenotype may be better understood within an imaging‐genetics framework rather than as an isolated morphologic entity.

From a genetic perspective, our data reinforce the marked heterogeneity of restrictive remodeling while also identifying recurrent genotype‐specific patterns. The high prevalence of reportable variants in this cohort suggests that a restrictive phenotype may be a genetically informative presentation (Gowda et al. 2022; Mogensen et al. 2015). Among the identified genes, MYBPC3 was the predominant genotype and appeared to define a subgroup with combined hypertrophic and restrictive features (Depasquale et al. 2012; Maron et al. 2002; Brodehl and Gerull 2022). TTR and LAMP2 variants were also observed in patients with overlapping phenotypes, highlighting the diverse molecular substrates that may converge on restrictive physiology. These findings suggest that the detection of restrictive features on imaging may support consideration of genetic testing in patients with otherwise ambiguous or mixed cardiomyopathy phenotypes (Chintanaphol et al. 2022).

Aortic sinus diameter was smaller in genotype‐positive than in genotype‐negative patients. This finding should be regarded as an exploratory observation rather than evidence of a causal mechanism. A smaller aortic sinus may reflect body size, loading conditions, age, or broader remodeling. Multivariable analysis in an adequately sized cohort would be required to establish an independent association or mechanistic link. Clinically, aortic sinus diameter should not be used alone to guide genetic testing or risk stratification.

The findings support consideration of genetic evaluation in patients with restrictive physiology, particularly when hypertrophic, storage or infiltrative, conduction, skeletal‐muscle, or familial features coexist (Muchtar et al. 2017; Yadav et al. 2019; Niss et al. 2016). Combining genetic information with echocardiographic phenotyping may improve recognition of clinically meaningful subgroups and refine diagnostic classification. Such an approach may ultimately support personalized surveillance, timely referral for advanced heart failure care, and the selection of patients for targeted therapies as the therapeutic landscape evolves (Yuan et al. 2017). However, given the limited number of patients, the adverse clinical courses observed in this study should be considered descriptive and require confirmation in larger longitudinal cohorts.

## 5 Limitations

This study has several limitations. First, its single‐center referral design may have enriched the cohort for severe, diagnostically complex, or genetically selected cases, resulting in selection and referral bias. The ethnicity of the participants, referral pathways, imaging practices, and tertiary‐care setting may limit generalizability to community populations, other ancestries, and unselected cardiomyopathy cohorts. Second, only 33 patients met the restrictive‐phenotype definition, genotype subgroups were small, and multiple correlated imaging comparisons were exploratory and were not adjusted for multiple testing; nominal associations may therefore include false‐positive findings. Third, follow‐up and longitudinal phenotyping were limited, and the small number of events precluded robust multivariable or gene‐specific survival analyses. Fourth, WES may miss deep intronic variants, structural variants, repeat expansions, mitochondrial variants, and other disease mechanisms. Finally, observational associations cannot establish mechanisms linking genotype, aortic dimensions, and restrictive remodeling. Multicenter, ancestry‐diverse cohorts with harmonized phenotyping and prospective follow‐up are needed.

## 6 Conclusions and Clinical Implications

In this single‐center cohort, P/LP variants were common among patients with cardiomyopathy and restrictive physiology, and exploratory genotype‐imaging comparisons identified differences in aortic sinus dimension and hypertrophic distribution. Integrating genetic testing with careful phenotyping may aid etiologic diagnosis, but the observed imaging and outcome associations require independent validation and should not yet be used as stand‐alone prognostic markers.

## Author Contributions

**Nianwei Zhou:** conceptualization, methodology, resources, writing – original draft, funding acquisition, writing – review and editing, validation. **Yi Sun:** software, data curation, methodology, investigation. **Xuemei Wei:** data curation, software, investigation. **Wenwen Liang:** software, formal analysis, supervision. **Fangmin Meng:** supervision, visualization. **Zhengdan Ge:** data curation, visualization. **Minmin Sun:** investigation, project administration, validation. **Xiaotong Cui:** project administration, resources. **Shuning Zhang:** resources, writing – review and editing. **Cuizhen Pan:** investigation, visualization. **Junbo Ge:** writing – review and editing, resources. **Xianhong Shu:** resources, funding acquisition, writing – review and editing, validation.

## Funding

This work was supported by National Natural Science Foundation of China, 82572239 and National Key Research and Development Program of China, 2024YFC3406702.

## Conflicts of Interest

The authors declare no conflicts of interest.

## Supporting information


**Table S1:** Genetic findings and follow‐up outcomes in patients with cardiomyopathy and a restrictive phenotype.
**Table S2:** Distribution of segmental hypertrophy among patients with a restrictive phenotype, stratified by genetic status.
**Table S3:** Baseline characteristics of patients with reportable genetic variants: MYBPC3 versus non‐MYBPC3 variants.

## Data Availability

The data that support the findings of this study are available on request from the corresponding author. The data are not publicly available due to privacy or ethical restrictions.
