# Supplementary material for: Genetic Insights Into Cardiomyopathy With Restrictive Phenotype: Implications for Diagnosis and Personalized Care
Source: Mol Genet Genomic Med. 2026 Oct 25;14(11):e70323. doi: 10.1002/mgg3.70323 (PMC13696024; doi:10.1002/mgg3.70323)
Supplement: Supplementary file 1 [file MGG3-14-e70323-s001.docx]

**Supplementary Table 1. Genetic findings and follow-up outcomes in patients with cardiomyopathy and a restrictive phenotype.**

| **Phenotype** | **Sex** | **Age (y)** | **Gene** | **Genomic position** | **Transcript** | **cDNA change** | **Protein change** | **ACMG evidence** | **Follow-up duration and outcomes** |
| --- | --- | --- | --- | --- | --- | --- | --- | --- | --- |
| RCM | Female | 71 | FLNC | 7:128494151 | NM_001458.5 | c.6509G>T | p.(Arg2170Leu) | PM2_supporting; PP3 | 5 years; chest tightness and orthopnea |
| RCM | Male | 66 | PLN | 6:118880229 | NM_002667.5 | c.145G>C | p.(Val49Leu) | PM2_supporting; PP3 | 5 years; exercise intolerance |
| RCM | Female | 72 | — | — | — | — | — | — | 1 year; atrial fibrillation, sudden cardiac death |
| RCM | Male | 30 | DES | 2:220286276 | NM_001927.4 | c.1238A>G | p.(Glu413Gly) | PM2_supporting; PP3 | 5 years; atrial fibrillation and lower-extremity edema |
| RCM | Male | 69 | PLN | 6:118880157 | NM_002667.5 | c.73C>G | p.(Arg25Gly) | PS4_supporting; PM2; PP1; PP3 | 3 years; died of heart failure |
| RCM | Female | 36 | ACADM; TNNT2 | 1:75761261; 1:201365298 | NM_001286043.2 NM_001001430.3 | c.1184G>A c.274C>T | p.(Gly395Glu) p.(Arg92Trp) | PS3; PM3_strong; PM2 PS3; PS4; PP1; PM5 | 3 years; sudden cardiac death following seafood-triggered diarrhea |
| RCM | Female | 35 | LAMP2 | X:120442598 | NM_002294.3 | c.928+1G>A | p.? (splice) | PVS1; PS4_supporting; PM2 | 1 year; heart transplantation |
| HRCM | Female | 27 | — | — | — | — | — | — | 5 years; chest tightness and orthopnea |
| HRCM | Male | 41 | FLNC | 7:128493805 | NM_001458.5 | c.6299G>A | p.(Arg2100His) | PM2_supporting; PP3 | 2 years; sudden cardiac death |
| HRCM | Female | 49 | MYBPC3 | 11:47365098 | NM_000256.3 | c.1153_1168del | p.(Val385MetfsTer16) | PVS1; PS4_moderate; PM2 | 1 year; ventricular tachycardia followed by unsuccessful resuscitation and death |
| HRCM | Male | 64 | MYBPC3 | 11:47364422 | NM_000256.3 | c.1415_1416del | p.(Glu472ValfsTer2) | PVS1; PM2 | 5 years; atrial fibrillation and chest tightness |
| HRCM | Male | 54 | MYBPC3 | 11:47353639 | NM_000256.3 | c.3798C>G | p.(Cys1266Trp) | PM2; PP3 | 5 years; chest tightness |
| HRCM | Male | 75 | LMNA | 1:156084717 | NM_170707.4 | c.8C>A | p.(Thr3Asn) | PM2_supporting; PP3 | 5 years; chest tightness and bradycardia |
| HRCM | Male | 50 | NEBL | 10:21139402 | NM_006393.3 | c.1037del | p.(Asn346IlefsTer46) | PM2; PVS1 not applied | 5 years; chest tightness |
| HRCM | Male | 69 | MYBPC3 | 11:47354209 | NM_000256.3 | c.3535G>A | p.(Glu1179Lys) | BS1_supporting; PP3 | 2 years; atrial fibrillation and death at home |
| HRCM | Male | 54 | — | — | — | — | — | — | 1 year; sudden cardiac death |
| HRCM | Male | 47 | MYBPC3 | 11:47360090 | NM_000256.3 | c.2288_2289insA | p.(Asn763LysfsTer70) | PVS1; PM2 | 5 years; atrial fibrillation and lower-extremity edema |
| HRCM | Male | 50 | TTR; TTR | 18:29172904; 18:29172905 | NM_000371.4 NM_000371.4 | c.115G>A c.116C>G | p.(Ala39Thr) p.(Ala39Gly) | PM2; PM5_supporting PM2; PM5_supporting | 5 years; frequent premature ventricular contractions |
| HRCM | Male | 30 | — | — | — | — | — | — | 3 years; sudden death at home |
| HRCM | Female | 51 | — | — | — | — | — | — | 2 years; death |
| HRCM | Female | 62 | TNNI3 | 19:55154097 | NM_000363.5 | c.481del | p.(Ala161ProfsTer?) | PVS1_strong; PM2; PP4 | 4 years; ventricular fibrillation and ICD implantation |
| HRCM | Male | 64 | TTR | 18:29172905 | NM_000371.4 | c.116C>A | p.(Ala39Asp) | PS4; PS3; PM2; PM5 | 2 years; died of heart failure |
| HRCM | Male | 51 | — | — | — | — | — | — | 5 years; atrial fibrillation |
| HRCM | Female | 17 | TNNT2 | 1:201328752 | NM_001001430.3 | c.819_820insA | p.(Val274SerfsTer9) | PVS1_strong; PM2 | 5 years; chest tightness |
| HRCM | Female | 66 | — | — | — | — | — | — | 5 years; chest tightness |
| HRCM | Male | 72 | MYBPC3 | 11:47354169 | NM_000256.3 | c.3575T>C | p.(Val1192Ala) | PM2_supporting; PP3 | 2 years; death at home |
| HRCM | Female | 32 | DES | 2:220285322 | NM_001927.4 | c.841T>G | p.(Tyr281Asp) | PM2; PP3 | 5 years; chest tightness |
| HDRCM | Male | 17 | LAMP2 | X:119581695 | NM_002294.3 | c.741+1G>T | p.? (splice) | PVS1; PS3; PS4_supporting; PM2 | 2 years; sudden cardiac death after a respiratory infection |
| HDRCM | Female | 34 | LAMP2 | X:119575596 | NM_002294.3 | c.1082del | p.(Lys361SerfsTer19) | PVS1; PM2; PP4 | 1 year; frequent premature ventricular contractions followed by sudden cardiac death |
| HDRCM | Male | 68 | DES; MYBPC3 | 2:220285006; 11:47360215 | NM_001927.4 NM_000256.3 | c.674_677dup c.2164G>T | p.(Glu226AspfsTer6) p.(Glu722Ter) | PVS1; PM2 PVS1; PS4_supporting; PM2 | 5 years; atrial fibrillation |
| HDRCM | Male | 68 | — | — | — | — | — | — | 5 years; atrial fibrillation |
| HDRCM | Male | 61 | — | — | — | — | — | — | 5 years; atrial fibrillation and orthopnea |
| HDRCM | Male | 66 | TNNI3 | 19:55663278 | NM_000363.5 | c.554G>A | p.(Arg185Gln) | PS3; PS4; PP1; PM2 | 5 years; chest tightness |

HRCM indicates hypertrophic cardiomyopathy with a restrictive phenotype; HDRCM, dilated cardiomyopathy with a restrictive phenotype

**Supplementary Table 2. Distribution of segmental hypertrophy among patients with a restrictive phenotype, stratified by genetic status.**

| **Characteristic** | **Entire cohort (n=33)** | **Genotype-negative (n=9)** | **Genotype-positive (n=24)** | **P value** |
| --- | --- | --- | --- | --- |
| Basal anterior (S1) | 22 (66.67) | 8 (88.89) | 14 (58.33) | 0.056 |
| Basal anteroseptal (S2) | 23 (69.70) | 8 (88.89) | 15 (62.50) | 0.093 |
| Basal inferoseptal (S3) | 23 (69.70) | 8 (88.89) | 15 (62.50) | 0.093 |
| Basal inferior (S4) | 21 (63.64) | 7 (88.89) | 14 (58.33) | 0.294 |
| Basal inferolateral (S5) | 21 (63.64) | 7 (77.78) | 14 (58.33) | 0.294 |
| Basal anterolateral (S6) | 21 (63.64) | 7 (77.78) | 14 (58.33) | 0.294 |
| Mid anterior (S7) | 21 (63.64) | 7 (77.78) | 14 (58.33) | 0.294 |
| Mid anteroseptal (S8) | 22 (66.67) | 7 (77.78) | 15 (62.50) | 0.423 |
| Mid inferoseptal (S9) | 22 (66.67) | 7 (77.78) | 15 (62.50) | 0.423 |
| Mid inferior (S10) | 21 (63.64) | 7 (77.78) | 14 (58.33) | 0.294 |
| Mid inferolateral (S11) | 21 (63.64) | 7 (77.78) | 14 (58.33) | 0.294 |
| Mid anterolateral (S12) | 21 (63.64) | 7 (77.78) | 14 (58.33) | 0.294 |
| Apical anterior (S13) | 20 (60.61) | 6 (66.67) | 14 (58.33) | 0.674 |
| Apical septal (S14) | 21 (63.64) | 6 (66.67) | 15 (62.50) | 0.831 |
| Apical inferior (S15) | 20 (60.61) | 6 (66.67) | 14 (58.33) | 0.674 |
| Apical lateral (S16) | 21 (63.64) | 6 (66.67) | 15 (62.50) | 0.831 |
| Basal (S1-S6) | 3.97 ± 2.79 | 5.00 ± 2.12 | 3.58 ± 2.95 | 0.143 |
| Mid-cavity (S7-S12) | 3.88 ± 2.87 | 4.67 ± 2.65 | 3.58 ± 2.95 | 0.325 |
| Apical (S13-S16) | 2.48 ± 1.92 | 2.67 ± 2.00 | 2.42 ± 1.93 | 0.745 |
| Anterior (S1 + S7 + S13) | 1.91 ± 1.42 | 2.33 ± 1.12 | 1.75 ± 1.51 | 0.242 |
| Anteroseptal (S2 + S8 + S14) | 2.00 ± 1.39 | 2.33 ± 1.12 | 1.88 ± 1.48 | 0.352 |
| Inferoseptal (S3 + S9) | 1.36 ± 0.93 | 1.67 ± 0.71 | 1.25 ± 0.99 | 0.194 |
| Inferior (S4 + S10 + S15) | 1.88 ± 1.45 | 2.22 ± 1.30 | 1.75 ± 1.51 | 0.388 |
| Inferolateral (S5 + S11) | 1.27 ± 0.98 | 1.56 ± 0.88 | 1.17 ± 1.01 | 0.294 |
| Anterolateral (S6 + S12 + S16) | 1.91 ± 1.42 | 2.22 ± 1.30 | 1.79 ± 1.47 | 0.427 |

Data are presented as n (%) or mean ± SD.

**Supplementary Table 3. Baseline characteristics of patients with reportable genetic variants: MYBPC3 versus non-MYBPC3 variants.**

| **Characteristic** | **MYBPC3 variant (n=7)** | **Non-MYBPC3 variants (n=17)** | **P value** |
| --- | --- | --- | --- |
| Age (years) | 60.43 ± 10.24 | 49.12 ± 18.85 | 0.142 |
| Male sex, n (%) | 6 (85.71) | 10 (58.82) | 0.231 |
| Body mass index (kg/m²) | 24.6 ± 8.1 | 24.0 ± 7.6 | 0.491 |
| Aortic sinus diameter (mm) | 31.86 ± 2.27 | 30.35 ± 3.84 | 0.380 |
| Left atrial diameter (mm) | 53.57 ± 8.85 | 47.64 ± 7.07 | 0.081 |
| LV end-diastolic diameter (mm) | 47.0 ± 4.62 | 48.24 ± 7.35 | 0.722 |
| LV end-systolic diameter (mm) | 33.71 ± 5.53 | 36.71 ± 8.51 | 0.429 |
| Anteroseptal wall thickness (mm) | 16.72 ± 3.04 | 11.24 ± 2.68 | 0.001 |
| Posterior wall thickness (mm) | 13.86 ± 2.79 | 11.35 ± 3.02 | 0.134 |
| Maximum LV wall thickness (mm) | 18.0 ± 3.27 | 12.24 ± 3.17 | 0.008 |
| RV hypertrophy, n (%) | 5 (71.43) | 7 (41.12) | 0.189 |
| PASP (mmHg) | 42.14 ± 12.29 | 44.53 ± 11.94 | 0.651 |
| LVEF (%) | 47.43 ± 9.16 | 46.53 ± 8.62 | 0.808 |
| TAPSE (mm) | 14.86 ± 2.41 | 12.35 ± 2.45 | 0.076 |
| Abnormal myocardial echogenicity, n (%) | 5 (71.43) | 7 (41.12) | 0.196 |
| LV systolic dysfunction, n (%) | 6 (85.71) | 16 (94.12) | 0.580 |
| RV systolic dysfunction, n (%) | 6 (85.71) | 16 (94.12) | 0.580 |
| E/e′ ratio | 19.1 ± 5.2 | 19.9 ± 6.0 | 0.418 |

Data are presented as mean ± SD or n (%). LV indicates left ventricular; RV, right ventricular; PASP, pulmonary artery systolic pressure; LVEF, left ventricular ejection fraction; and TAPSE, tricuspid annular plane systolic excursion.
